# Correlations Between the DMN and the Smoking Cessation Outcome of a Real-Time fMRI Neurofeedback Supported Exploratory Therapy Approach: Descriptive Statistics on Tobacco-Dependent Patients

**DOI:** 10.1177/15500594211062703

**Published:** 2021-12-08

**Authors:** Marco Paolini, Daniel Keeser, Boris-Stephan Rauchmann, Sarah Gschwendtner, Hannah Jeanty, Arne Reckenfelderbäumer, Omar Yaseen, Paul Reidler, Andrea Rabenstein, Hessel Jan Engelbregt, Maximilian Maywald, Janusch Blautzik, Birgit Ertl-Wagner, Oliver Pogarell, Tobias Rüther, Susanne Karch

**Affiliations:** 1 9183 Department of Radiology, University Hospital, LMU Munich, Munich, Germany; 2 9183 Department of Psychiatry and Psychotherapy, University Hospital, LMU Munich, Munich, Germany; 3 Hersencentrum Mental Health Institute, Amsterdam, the Netherlands; 4 548909 Institute for Radiology and Nuclear Medicine St. Anna, Luzern, Switzerland; 5 Division of Neuro-Radiology, The Hospital for Sick Children, University of Toronto, Toronto, Canada

**Keywords:** resting-state functional connectivity, real-time fMRI, neurofeedback, tobacco dependence, smoking cessation

## Abstract

The aim of this study was to explore the potential of default mode network (DMN) functional connectivity for predicting the success of smoking cessation in patients with tobacco dependence in the context of a real-time function al MRI (RT-fMRI) neurofeedback (NF) supported therapy.

Fifty-four tobacco-dependent patients underwent three RT-fMRI-NF sessions including resting-state functional connectivity (RSFC) runs over a period of 4 weeks during professionally assisted smoking cessation. Patients were randomized into two groups that performed either active NF of an addiction-related brain region or sham NF. After preprocessing, the RSFC baseline data were statistically evaluated using seed-based ROI (SBA) approaches taking into account the smoking status of patients after 3 months (abstinence/relapse).

The results of the real study group showed a widespread functional connectivity in the relapse subgroup (n = 10) exceeding the DMN template and mainly low correlations and anticorrelations in the within-seed analysis. In contrast, the connectivity pattern of the abstinence subgroup (n = 8) primarily contained the core DMN in the seed-to-whole-brain analysis and a left lateralized correlation pattern in the within-seed analysis. Calculated Multi-Subject Dictionary Learning (MSDL) matrices showed anticorrelations between DMN regions and salience regions in the abstinence group. Concerning the sham group, results of the relapse subgroup (n = 4) and the abstinence subgroup (n = 6) showed similar trends only in the within-seed analysis.

In the setting of a RT-fMRI-NF-assisted therapy, a widespread intrinsic DMN connectivity and a low negative coupling between the DMN and the salience network (SN) in patients with tobacco dependency during early withdrawal may be useful as an early indicator of later therapy nonresponse.

## Introduction

Therapeutic strategies for the treatment of tobacco disorder and addiction even include various types of biofeedback and neurofeedback (NF) procedures^1‐3^. The innovative technique of real-time functional MRI (RT-fMRI) NF enables individualized, target-oriented neuromodulation training of brain areas and brain networks for voluntary control with high spatial accuracy even in deep subcortical brain areas. fMRI is based on a neurovascular coupling principle^4^ and the blood oxygenation level-dependent (BOLD) effect^5^. RT-fMRI has become increasingly available and applicable within the last decade, leading to an increasing number of patient studies with promising results, particularly in the field of neurological and psychiatric disorders^6‐13^. In patients with tobacco use disorder, RT-fMRI-NF has been successfully applied for modulation of cue-related neural responses with different impact on craving behavior^14‐19^.

The application of RT-fMRI-NF can also have significant effects on fMRI-based brain connectivity known to be altered in various neuropsychiatric disorders including addiction and tobacco use disorder^20,21^. In principle, functional connectivity is characterized by correlations of low frequency fluctuations in the BOLD signal between brain regions and can be measured in the resting state^22^. This baseline activity of the brain has revealed different consistent functional networks^23‐26^.

Addiction related diseases have been shown to influence functional brain connectivity. Especially the executive control network (ECN), the default mode network (DMN) and the salience network (SN) are affected in addiction as well as their coupling^21,27,28^. Core regions of the DMN, the main network of internal-focused processing, are the medial parietal cortex (MPL), the medial prefrontal cortex (MPFC), the temporoparietal junctions (TPJs) and the hippocampi^29‐31^; those of the ECN related to extrinsic attention are the dorsolateral prefrontal cortex (DLPFC) and the posterior parietal cortex (PPC)^23,32^. The SN is primarily responsible for switching between the DMN and the ECN and mainly consists of the anterior cingulate cortex (ACC) and the anterior insula of both hemispheres^32,33^. Due to the effects of nicotine, chronic consumption of tobacco mainly leads to different disruption of these networks, largely demarcated during abstinence. In the state of tobacco withdrawal, attention is focused on internal DMN-related processing away from ECN-related external stimulus processing^21,28^. In a seed-based study, the DMN and its counterpart, the ECN, have shown opposite shifts in functional connectivity in abstinent smokers towards different subdivisions of the insula^34^. In another recent study, in the early state of abstinence, an increasing influence of the DMN was observed and an altered network coupling, especially between the DMN and SN, was associated with a lower task performance and increased smoking urges^27^. Moreover, during abstinence reduced functional connections between the insula and control-related and sensorimotor-related brain regions like the dACC and the DLPFC seem to play an important role in smoking cessation and therapy outcome^35‐37^.

Overall, due to their central role in addiction, the DMN was identified as promising target for developing functional biomarkers^38^. In different substance use disorders, the DMN was shown to be associated with psychological well-being, ruminations, craving, and clinical outcome based on altered cognitive and emotional processing^38‐41^. Furthermore, an improvement of RT-fMRI-NF training with impact on brain connectivity and cigarette craving was described using DMN-dependent NF signals.^18^ Since characteristic changes in DMN-functional connectivity of chronic smokers are unmasked in the state of withdrawal and taking in account that RT-fMRI-NF may have a primary or secondary influence to certain brain regions, the initial situation of brain connectivity in the early state of abstinence before applying NF seems to contain the most valuable and reliable information^21,27,28^.

In our recent RT-fMRI-NF study on tobacco-dependent patients, we have described correlations between therapy outcome and BOLD signal changes in addiction-related brain regions during the NF training^17^. Our results were based on the neural responses of smokers grouped by their smoking status (abstinence/relapse) three months after completing a certified smoke-free course. The course included an additional experimental RT-fMRI-NF training in the early abstinence state. In this study report, we had not included any postprocessing or statistical analyses of functional connectivity data. In particular, the purpose of the current study report was to investigate the potential of baseline resting-state functional connectivity (RSFC)-MRI associated with therapeutic outcome in the early withdrawal. In addition, the results of the control (sham) NF group were also included. To our knowledge, this is the first study on patients with tobacco disorder to investigate the relationship between DMN-related functional connectivity and the outcome of smoking cessation in early withdrawal of a RT-fMRI-NF supported exploratory therapy approach.

## Materials and Methods

### Subjects

The patient cohort (n = 54) consisting of 22 females and 32 males was the same as described in Karch et al 2019. The main inclusion criteria were age between 18 and 65 years, ICD-10 diagnosis of nicotine dependence (F17.2) without the existence of other neurological or psychiatric lifetime diagnoses, no prior head injury and the lack of MRI contra-indications. At the time of the study testing, all participants acknowledged a solid mental and physical constitution. The whole psychotherapeutic program was free of charge. For participating the fMRI scans, patients were paid 50€ per session. Approval was given from the local research ethics committee of the Medical Faculty of LMU Munich and the study is in accordance with the Declaration of Helsinki. Taking into account the same exclusions as described in Karch et al 2019^17^ plus further exclusions due to deficient data and extensive head motion during the resting-state scans (n = 8), results of 28 tobacco-dependent smokers (10 females, 18 males) were included in the functional connectivity analysis, either in the study group (real group  =  RG, n = 18) or in the control group (sham group  =  SG, n = 10).

### Study Design

Patients took part in an established and certified psychotherapeutic group program developed for quitting smoking^42^ based on group sessions and various techniques for behavioral change. As experimental therapeutic add-on, three scanning sessions including RT-fMRI-NF-training were performed during this program after all patients had stopped smoking simultaneously on a predefined day, ie in the state of withdrawal. We focused on the resting-state measurements of the first resting-state run of the first session acquired after a cue localizer paradigm as it was the only run that was prior the beginning of the whole NF training procedure. A single brain region within the ACC, the DLPFC, or the insula that was individually determined by the localizer scan in each session was used as NF target area. It was defined as the cluster with the most extensive BOLD response within these addiction-related regions in order to train always the currently most relevant functional area during therapy. Using a graphical bar, the patients should downregulate addiction-related BOLD -signal in these areas without any predefined strategies. For sham feedback, a brain region was selected that had not been involved in the addiction-related neural response of the localizer scan. Further information about the study design, the smoking-free course and the NF training are described in detail in Karch et al 2019.^17^ Sociodemographic and psychopathological data as well as information about smoking and craving were collected by standardized questionnaires.

### Acquisition and Analysis of Clinical Data

Besides the collection of sociodemographic data, symptom severity and other psychometric aspects were assessed by questionnaires before and after scanning: The Fagerström Test for Nicotine Dependence (FTND)^43^, the Questionnaire on Smoking Urges – German (QSU-G)^44^, the Verbal Intelligence Test (WST)^45^, the Barratt Impulsiveness Scale (BIS-11)^46^, the Aggression Questionnaire (AQ)^47^, the Beck Depressions Inventory (BDI)^48^, the State-Trait Anger Expression Inventory (STAXI)^49^, the State-Trait Anxiety Inventory (STAI)^50^, and the NEO-Five-Factor Inventory (NEO-FFI)^51^. CO levels were measured via UBLOW CO breath tester (Neomed Medizintechnik GmbH).

Statistical analysis of the questionnaire ratings of all four patient subgroups, ie abstinent and relapse patients of both the RG and the SG, were calculated with SPSS version 25 with a level of significance *p* < .05. Due to the small sample size, we calculated the nonparametric Kruskal–Wallis test for independent samples over all subgroups, with a Dunn–Bonferroni *post hoc* test. For comparison of the relapse and abstinence rates, respectively, of the two groups SG and RG a chi-square test was calculated.

### Imaging and Analysis of Resting-State Functional Connectivity-MRI data

Images were acquired using a 3 T standard clinical scanner (Ingenia, Philips Healthcare) with a 32ch phased array head coil. Resting-state functional imaging was performed with a BOLD sensitive echo-planar gradient-echo sequence in axial orientation covering the whole brain using the following parameters: field of view (FOV), 240  ×  240  ×  147 mm; voxel size, 3 mm isotropic; imaging matrix, 80  ×  80; time of repetition (TR), 2500 ms; time of echo (TE), 30 ms; flip angle (FA), 90°; number of volumes, 180. Patients were instructed not to move, to keep their eyes closed without falling asleep and not to think of anything in particular. For anatomical reference, a high-resolution T1-weighted three-dimensional sequence was obtained in sagittal orientation with the following imaging parameters: FOV, 240  ×  220  ×  200 mm; voxel size, 1 mm isotropic; TR, 8.2 ms; TE, 3.8 ms; FA, 8°, number of slices.

#### Preprocessing of Resting-State Functional Connectivity-MRI data.

Our analysis was restricted to the baseline RSFC-MRI data acquired before NF training. The data sets were pre-processed as described in detail previously^52^. The only difference was that we used Advanced Normalization Tools (ANTs)^53^ instead of BET (FSL) for brain extraction. As head motion can significantly affect resting-state studies, we calculated head motion of each subject for the mean absolute and relative displacement (in mm) of each brain volume as compared to the previous volume. The translation parameters in *x*, *y*, and *z*-directions across all time were estimated for groups and subgroups separately. Motion exceeding a framewise displacement (FD) > 0.4 mm was censored and regressed out using this information as confounders^54^. This liberal FD threshold was chosen due to the small sample. Thresholds for complete exclusions were a mean FD>0.5 and a mean DVARS>3.0^54^. Group comparisons were calculated analogous to the clinical data in SPSS.

#### Statistical Analysis of Resting-State Functional Connectivity-MRI data.

**Figure 1.**
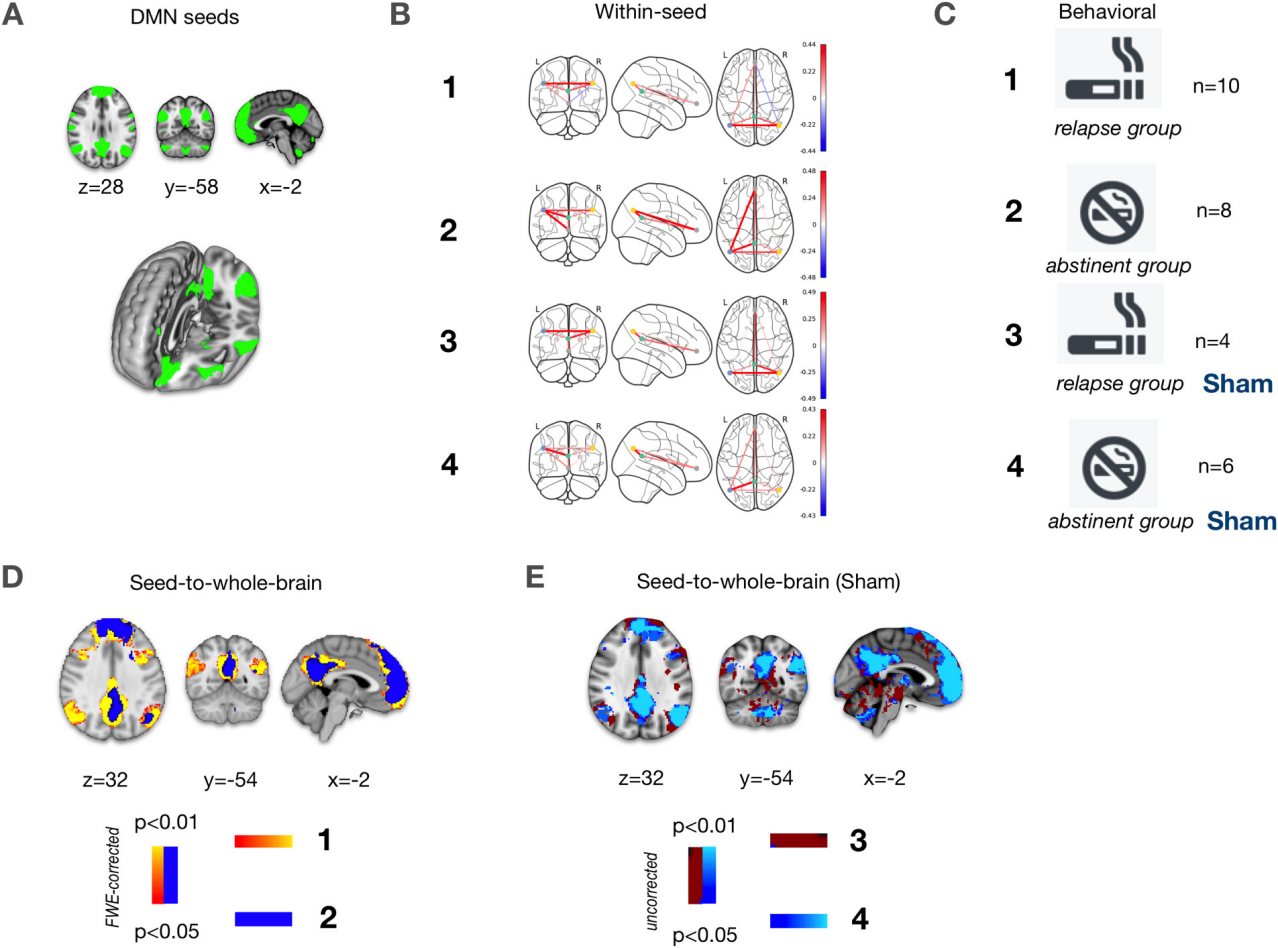
Seed definition and SBA results based on the DMN template of the UK Biobank. (A) Illustration of DMN seeds. (B) Group results of the DMN within-seed analysis showing correlations. (C) Legend for the definition of subgroups. (D/E) Group results of the DMN seed-to-whole-brain analysis showing correlations for the real study group/sham control group.

Abbreviations: DMN: default mode network; SBA: seed-based ROI approach.

**Figure 2.**
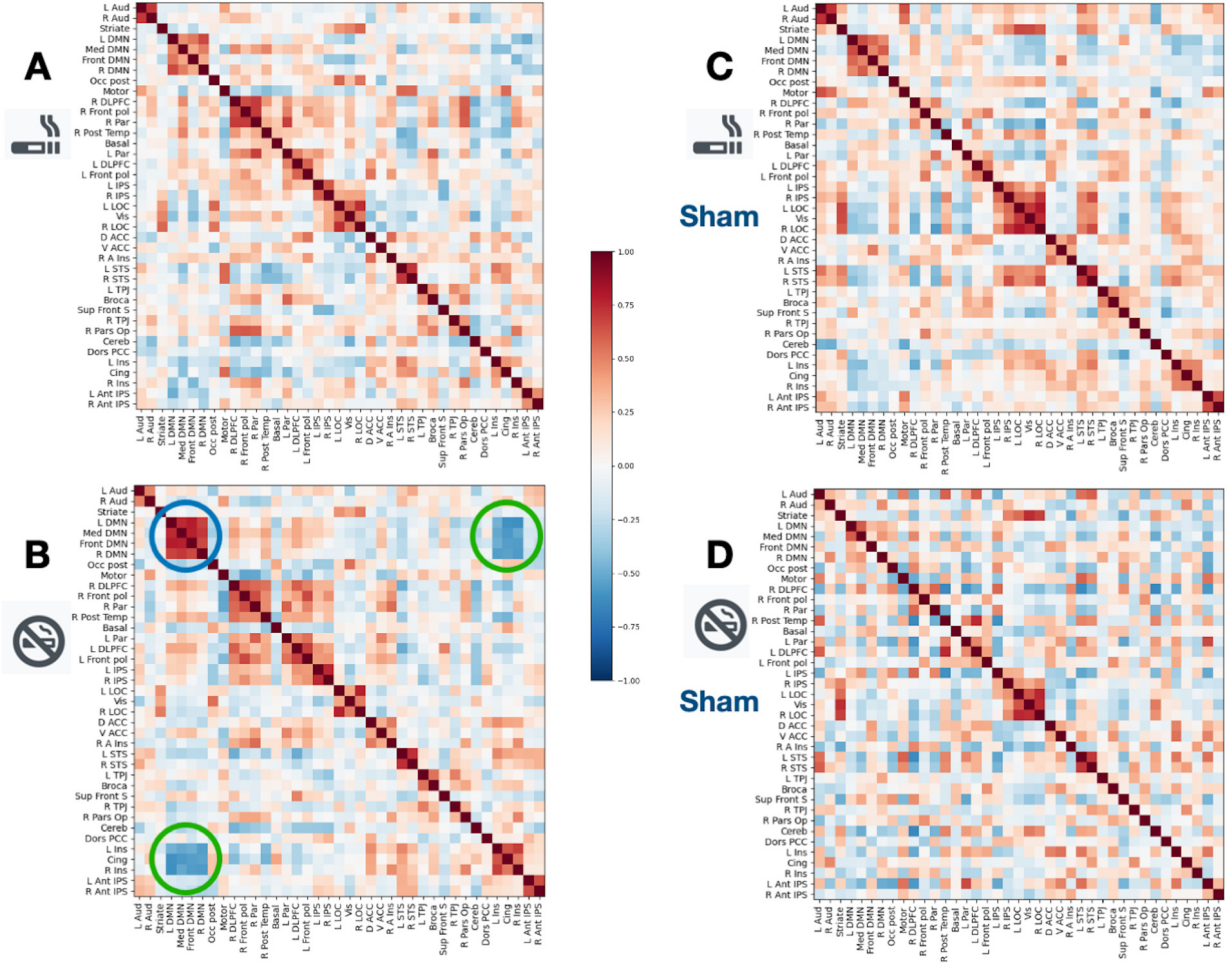
Connectivity matrices based on the Multi-Subject Dictionary Learning (MSDL) atlas. Heat maps are shown for the relapse subgroup (A) and the abstinent subgroup (B) of the real study group as well as for the relapse subgroup (C) and the abstinent subgroup (D) of the sham control group. In (B), the blue circle marks high within DMN seed correlations (red), the green circles strong DMN anticorrelations (blue) to the insular brain regions and to the cingulate cortex.

Abbreviations: Aud: auditory; ACC: anterior cingulate cortex; Basal: basal ganglia; Cereb: cerebellum; Cing: cingulum; DMN: default mode network; DLPFC: dorsolateral prefrontal cortex; Ins: insula; IPS: intraparietal sulcus; LOC: lateral occipital cortex; Pars Op: pars opercularis; PCC: posterior cingulate cortex; .STS: Superior Temporal Sulcus; TPJ: temporoparietal junction; Vis: visual.

Single-subject RSFC-MRI data were processed using a seed-based ROI approach (SBA). As ROI we used the DMN template of the UK Biobank (Figure 1A) covering 19,830 subjects (http://biobank.ctsu.ox.ac.uk/crystal/field.cgi?id=25754).^55^ Voxelwise within-group SBA statistics were calculated using Randomise2.9 implemented in FSL 6.0 (https://fsl.fmrib.ox.ac.uk/fsl/fslwiki/). We considered effects significant at a family-wise error (FWE) corrected and Threshold-Free-Cluster-Enhancement (TFCE) pcorr<0.05 value in the RG. Due to the small sample size, also uncorrected results were taken into account in the sham group for detecting trends (*p* < .05). Negative and positive correlations were calculated from the DMN seed to all other voxels in the whole brain using fslmaths to extract voxels and R3.4.1 was used for descriptive statistics. For the positive and negative correlations, we used ten averages of the individual fcMRI data from each group. For the visualization of individual DMN-maps we used Nilearn^56^ and as MNI coordinates for the DMN: PCC (0, −52, 18), left TPJ (−46, −68, 32), right TPJ (46, −68, 32), and MPFC/ACC (1, 50, −5). There were only marginal overlaps between our ROIs and the UK Biobank DMN template seeds—containing parts of the rostral and genual ACC. Moreover, the Multi-Subject Dictionary Learning (MSDL) atlas^57^ was used to determine the association of DMN regions with other parts of the brain containing DMN nodes outside the NF target areas. For this purpose, a connectivity matrix was calculated and visualized (Figure 2).

## Results

### Clinical Data

Between-group and between-subgroup comparisons of collected clinical data were calculated. In our RG, 3 months after smoking cessation, 8 patients (females [f], 12.5%; mean age [ma], 46.5  ±  10.1; right-handed [rh], 87.5%; pack years [py], 30.8  ±  12.7) were still abstinent, whereas 10 patients (f, 40%; ma, 36.20  ±  11.3; rh, 80%; py, 17.6  ±  13.8) had a relapse. The abstinence rate was 44.4%. In the SG, six patients (f, 50%; ma, 42.0  ±  12.6; rh, 80%; py, 27.3  ±  15.1) remained abstinent and four patients (f, 50%; ma ± 56.0  ±  8.6; rh, 75%; py, 34.5  ±  14.8) relapsed. The abstinence rate was 60%. The difference between the RG and SG was not significant regarding abstinence or relapse rates (χ^2^: abstinent: *p* = .637; relapse: *p *= .527). The comparison of patients of the RG and SG showed no significant difference in the numbers of pack years or any psychometric test. The comparisons of the four subgroups showed a significant difference regarding the anger-in subscale of STAXI (*p* = .003). *Post hoc* statistics revealed a significant difference between the abstinence subgroup and relapse subgroup of the RG (*p* = .039), and between the relapse subgroup of the RG and the abstinent subgroup of the SG (*p* = .048). All other questionnaires did not show any significant difference.

### Functional Connectivity Data

#### Head Motion

Head motion, measured by the mean relative displacement (in mm), revealed an average of 0.12  ±  0.09 (RG) and 0.23  ±  0.13 (SG). In the RG, the displacements were 0.13  ±  0.08 (relapse subgroup) and 0.11  ±  0.10 (abstinence subgroup). In the SG, the displacements were 0.29  ±  0.15 (relapse subgroup) and 0.18  ±  0.10 (abstinence subgroup). There were no significant between-group differences (*p* < .05).

#### Connectivity Matrix Calculation

The MSDL connectivity matrices are illustrated as heat maps for both groups in Figure 2. For the abstinent group of the RG, the within-DMN seed correlations and the DMN seed anticorrelations to the insular brain regions and to the cingulate cortex were homogenously high (Figure 2B, green circles and blue circles). For the corresponding relapse group, no equivalent pattern is demarcated in the matrix (Figure 2A). In the SG, no comparable effect was found between subgroups (Figure 2C and Figure 2D) by visual inspection.

#### Seeb-Based ROI FC-MRI using the UK Biobank Default Mode Network template

Within-seed correlations are presented correlative, as shown in Figure 1B for each group and in the supporting information figure for all individual subjects for the relapse and abstinent subgroups of each group. Overall, in the RG, the abstinent subgroup showed high positive within-DMN correlations with a left lateralized pattern, ie hardly involving the right TPJ node, whereas in the relapse subgroup a high positive correlation was only recognizable between both TPJs. The subgroups of the SG demonstrated similar within-DMN correlation patterns including the left lateralized configuration of the abstinent subgroup.

The seed-to-whole-brain group results for the relapse subgroups and the abstinent subgroups of the RG are shown in Figure 1D. In the RG, the relapse subgroup showed a DMN pattern with several significant correlations (*p* < .95, FWE-corrected) to frontal and temporal brain regions including bilateral insular regions exceeding the typical core nodes of the DMN. In contrast, the DMN of the respective abstinent subgroup was mainly restricted to core nodes, ie the PCC and the mPFC as well as small temporoparietal areas. In the SG, the resulting DMN seed-to whole-brain correlations of the subgroups (*p* < .95, FWE-corrected) hardly differed in extension by visual inspection (Figure 2E).

## Discussion

In the state of early abstinence, tobacco-dependent patients are thought to have a shift in functional brain connectivity towards intrinsic processing as a correlate of the enhanced influence of internal symptoms of withdrawal implicated by a strengthening of the DMN and a suppressed DMN-SN coupling^21,27,28^. Based on a placebo-controlled double blind study design, we found some evidence that these functional connectivity differences may be more defined and partially more pronounced in patients with future relapse compared to continuously abstinent patients in the context of a therapy supported by RT-fMRI-NF training. Our main finding was the association of negative therapeutic outcome with a broad appearing DMN and a prominent interaction of the DMN with other brain regions including main SN nodes implicating a potential predictive value of functional connectivity in tobacco-dependent patients in the state of early abstinence.

### Specificity of Functional Results and Potential Confounding

Apart from the within-seed results showing similar between-subgroups results in the RG and the SG, our observations seem to be specific for a therapy that is substantially based on RT-fMRI-NF training in cue-sensitive brain areas. Besides, different atlas-based analyses contributed to the specificity of our results. Furthermore, connectivity analysis effects cannot be attributed to head motion that was comparably small in groups and subgroups, unfortunately at cost of exclusions. Regarding confounding, a critical point is the influence of sociodemographic data, the severity of symptoms and the other psychometric information, especially in a small sample size^58‐62^. Indeed, this is the most crucial limitation of our study and certain effects cannot be ruled out. Significant differences between subgroups were found regarding the anger-in subscale of STAXI indicating a possible slightly dissimilar attitude of processing negative emotions. It was not exclusively found between subgroups of the RG as a hint for an unspecific observation.

### Default Mode Network-FC and Influence of Real-Time-fMRI-Neurofeedback during Withdrawal

The results of the DMN seed-based resting-state analysis of the RG demonstrated a broad connectivity pattern in the relapse subgroup exceeding the UK Biobank DMN template in frontal, parietal and temporal brain areas including the insular cortex, while the abstinent subgroup revealed a midline-oriented pattern. The latter pattern mainly contained the midline core subsystem of the DMN consisting of the PCC and anterior MPFC related to self-relevant processing and emotional decision-making^29,30^. This may indicate that functional connectivity associated with the intrinsic DMN is more widespread in patients with a higher risk for relapse. This is in line with prior knowledge about an enhanced DMN-connectivity to subcortical regions accounting for failures of cognitive and emotional control processing in people with substance use disorder^38,63^. The MSDL connectivity matrices that calculate other network ROIs further support these observations. In the RG high positive correlations were revealed between the DMN regions and regions of a cingulate-insular network (green circles in Figure 2B) that includes the brain areas of the classical SN^32^ thought to be related to the nicotine deprived state and withdrawal symptoms^27,28^. Instead, the abstinence subgroup showed high negative DMN-SN correlations and high positive within-DMN correlations presumably representing a state of intact coupling. A potentially assumable linkage between the number of ACC/insula NF target areas and therapy outcome was not provable as every subject of the RG (abstinent/relapse) trained at least in one session the right/left insula/ACC, and even the fraction of the overall ACC/insula ROIs were comparable between groups with 9% more in the RG. However, in general, dependences between the NF target areas and the connectivity networks including these areas may be possible.

According to the results of the within-seed analyses, a left lateralized configuration of the DMN with a low RTPJ coupling seems to be advantageous for the remaining smoking abstinent compared to a DMN pattern mainly characterized by a positive coupling between both TPJs. While the left TPJ is particularly involved in the different levels of language processing, the RTPJ is known to be a higher association area representing a linkage between external stimulus-related processing and internal processing^64,65^. The low appearing coupling of the RTPJ within the DMN in the abstinence subgroup of the RG may implicate a minor influence of external stimulation to intrinsic processing and vice versa, ie for example. little effect of smoking cues on the urge to smoke, and, on the other side, low craving interfering attention. However, corresponding results of the within-seed analyses of the RG and the SG were similar presumably indicating a not NF-specific observation and more general significance. At this point, also the unspecific and marginally significant difference regarding the anger in subscale within the RG has to be mentioned, as discussed in Section 4.1, as well as the observation that brain connectivity within the DMN may also be associated with stress or stress-like neural responses. These are known to arise in the state of early withdrawal^66^ and seem to be possible related to negative emotions. Different types of stress appear to influence RSFC, especially in the DMN^67,68^. A recent study showed clinical effects of biofeedback via skin temperature training concerning the degree of nicotine dependence and the degree of psychiatric symptoms as well as slight connectivity changes related to certain DMN nodes in smokers^69^.

The high relevance and the global effect of the TPJ coupling are specifically known in regard to the DMN. A stronger coupling of the anterior part of the RTPJ as well as the LTPJ with other DMN nodes is associated with connectivity changes in global networks including DMN-SN connectivity^65^. The simultaneous changes of within-network FC and between-network FC are also in line with the fc alterations described in the latter paragraphs. Moreover, the method of NF training itself may be affected by preexisting deficits of external attention triggered by alterations of the DMN and of its inter-network connectivity^38^. In that context, in addition, the right anterior insula is known to play a central role in the underlying neural mechanism of RT-fMRI-NF^70^. Taken the insula-related connectivity of our results into account, NF- associated functions of the right insula may be specifically affected in the relapse group and may have contributed to patients’ outcome.

### Limitations and Future Perspectives

Several limitations apply to our study, especially due to its exploratory nature. Specifically, the statistical analysis of clinical and functional data was limited by the small sample size of the subgroups and potential confounder as mentioned in paragraph 4.1. Besides, we did not perform direct between-group comparisons. Therefore, our results are descriptive and do not represent significant differences between groups. Otherwise, our functional imaging results are coherent among each other and in consent with prior knowledge indicating the need for future studies with larger sample sizes^21,28,38,71^. Furthermore, there are limitations due to the restriction associated with the methods of our analyses. We used predefined ROIs as described in the method section. However, the definition of resting-state networks is not completely uniform and static but variable and continuously developing. Nevertheless, our main conclusions were based on the well-known DMN and its core regions^29‐31^. Moreover, we used analyses based on different atlases and ROIs leading to concordant results. An additional critical point that has to be considered when interpreting our results is the whole study design containing a localizer run that was performed immediately before the resting-state run^17^. The impact of pre-scan tasks and activities on functional connectivity due to enduring memory and learning effects was previously described^72,73^. Therefore, persisting influence of the smoking cues presented in the localizer run on neural responses is possible. Indeed, in an fMRI study with a comparable setting, between-group connectivity differences based on smoking cessation outcome were discussed on a cue dependent background that was also considered as a limitation^74^. However, persisting cue influence could have been helpful for the demarcation of specific connectivity. Another limitation is that findings could not be generalized and have to be considered against the background of our specific setting including the experimental activity-based NF training and to the non-medicamentous cognitive behavioral therapeutic program^17^. In that context, identifying patient-specific therapeutic procedures and suitable time points for their application could be decisive for the final therapy outcome. Taking into account that in our study RT-fMRI-NF training had no significant impact on the smoking cessation rate, a consequence might be that our treatment procedure should be restricted to patients with an advantageous functional connectivity pattern representing a kind of precondition or a functional indicator. Otherwise, it has to be emphasized that despite the prospect of functional predictors the patient's clinical condition, ie the subjective feelings, the personal development during group therapy and the relationship to the therapist has to be considered primarily for therapy decision, and that the technique of RT-fMRI-NF focusing only on brain function may just support the patient's main therapy plan as an add-on therapy option. Future therapy concepts may contain RT-fMRI-NF training specific to the patient's clinical condition.

In conclusion, our results are consistent with several recent studies revealing that the knowledge about altered RSFC in tobacco-dependent patients offers a promising opportunity for a more target-oriented diagnostic monitoring during treatment after smoking cessation. In the context of our specific therapy program supported by activity-based RT-fMRI-NF of smoking cue-related brain areas, the organization of the DMN in regard to its subsystems and the interaction between the DMN and regions of the SN seem to play a crucial role in therapy outcome representing possible biomarkers during withdrawal. Future studies may prove our descriptive results with further analyses on larger sample sizes and may use more selective and target-oriented RT-fMRI-NF specific to the patient's individual clinical condition.
